# Association of EPCR Polymorphism rs867186-GG With Severity of Human Malaria

**DOI:** 10.3389/fgene.2020.00056

**Published:** 2020-02-24

**Authors:** Juan Carlos Cespedes, Jacqueline Hibbert, Sri Krishna, Fengxia Yan, Praveen K. Bharti, Jonathan K. Stiles, Mingli Liu

**Affiliations:** ^1^ Department of Microbiology, Biochemistry and Immunology, Morehouse School of Medicine, Atlanta, GA, United States; ^2^ National Institute for Research in Tribal Health (NIRTH), Jabalpur, India

**Keywords:** cerebral malaria, endothelial protein C receptor, activated protein C, pathogenesis, polymorphism

## Abstract

**Background:**

Cerebral malaria (CM) is characterized by the sequestration of *Plasmodium*-infected erythrocytes (pRBCs) to host brain microvasculature beds *via P. falciparum* erythrocyte membrane protein 1 (PfEMP1). Under normal conditions, activated protein C (APC) bound to endothelial protein C receptor (EPCR) has cytoprotective properties *via* the activation of protease-activated receptor 1 (PAR1). During malaria infection, pRBCs transports PfEMP1 to the membranes to bind EPCR in the same region as APC. As a result, APC is less capable of inducing cytoprotective effects *via* PAR1. Two studies involving adult malaria patients revealed that EPCR rs867186-GG allele is associated with protection against severe malaria, while three other studies involving child malaria patients could not show association between EPCR rs867186-GG genotype and severe malaria or increased mortality among children with CM.

**Methods:**

We examined the association between the EPCR rs867186-GG genotype and the protection against cerebral malaria. Peripheral blood samples were collected from 47 malaria patients and 34 healthy individuals from a study conducted from 2004 to 2007 at the NSCB Medical College Hospital in India. CM and malaria-associated complications were defined based on WHO criteria. Genomic DNA was isolated from the peripheral blood mononuclear cells. Primer sequences were designed to contain rs867186 of the *PROCR* gene (NM 006404) and were used to amplify a 660 bp product as described before. PCR products were purified, and DNA sequences were determined by Sanger Sequencing (Genewiz, NJ). Nonparametric tests were used to compare the groups. To analyze differences in allele frequencies, we used chi-squared or Fisher's exact tests for categorical variables if the expected values were less than 5. P-value <0.05 was considered statistically significant.

**Results:**

Our results showed significantly higher rates of AG and GG genotypes in CM patients compared to mild malaria (P = 0.0034).

**Conclusion:**

Our results indicate that rs867186-GG or rs867186-AG genotypes are not associated with protection against HCM.

## Background

Cerebral malaria (CM) is characterized by the sequestration of parasitized red blood cells (pRBC) to host brain microvasculature beds *via P. falciparum* erythrocyte membrane protein 1 (PfEMP1) (Nielsen et al., 2002; Jensen et al., 2004; Bull et al., 2005; Kyriacou et al., 2006; Rottmann et al., 2006; Moxon et al., 2011; Lavstsen et al., 2012). PfEMP1 protein is encoded by approximate 60 *var* genes with each parasite expressing only one *var* gene at a time (Scherf et al., 2008). The cysteine-rich interdomain region (CIDR) and Duffy binding-like (DBL) domains (Mkumbaye et al., 2017; Shabani et al., 2017; Tuikue Ndam et al., 2017) are located at the N-terminal of the extracellular portion of PfEMP1. The binding of PfEMP1 to different host receptors determines the amount of sequestration and is associated with disease severity (Newbold et al., 1997; Wassmer et al., 2004; Ochola et al., 2011). PfEMP1 has been found to bind to CD36 on platelets *via* CIDRα2-6 domains in uncomplicated/mild malaria (UM/MM) (Mkumbaye et al., 2017; Mustaffa et al., 2017), and to ICAM-1 on endothelial cells in severe malaria (SM) *via* DBLβ5 domains (Mustaffa et al., 2017; Tuikue Ndam et al., 2017). Recently, PfEMP1was found to interact with EPCR through CIDRα1 of domain cassette 8 (DC8) and DC13 in SM (Turner et al., 2013). By competing with activated protein C (APC), PfEMP1-CIDRα1 binding to EPCR induces pro-apoptosis, pro-inflammation, coagulation, compromises cytoprotection and barrier functions of EPCR (Bernabeu and Smith, 2017; Lennartz et al., 2017; Pendurthi and Rao, 2018), and leads to severe malaria including CM and severe malarial anemia (SMA) (Mosnier and Lavstsen, 2016). Shabani et al. examined PfEMP1 *var* genes transcripts of Ugandan child CM patients by RT-PCR and found that children with CM and SMA expressed higher EPCR-binding PfEMP1 transcripts, where EPCR-binding PfEMP1 transcripts were associated with severity of malaria (Shabani et al., 2017). The abundance of EPCR-binding CIDR α1 transcripts increased with malaria severity, which indicates that EPCR could be a novel therapeutic target for severe malaria. To deplete *P. falciparum* sequestration in host blood circulation, particularly in brain blood circulation, all EPCR-binding CIDRα1 subtypes are required to be targeted (Mkumbaye et al., 2017). Soluble EPCR (sEPCR) can competitively bind to the CIDRα1 domain, which frees EPCR binding sites for APC and restores EPCR cellular function. Plasma EPCR levels correlate with the genetic polymorphisms of the *EPCR* gene, in particular, the EPCR rs867186-GG genotype (Naka et al., 2014; Schuldt et al., 2014; Hansson et al., 2015; Moussiliou et al., 2015; Shabani et al., 2016). Two studies involving African and Thai adult malaria patients revealed that EPCR rs867186-GG genotype is associated with protection against severe malaria (Naka et al., 2014; Shabani et al., 2016), while three other studies (Schuldt et al., 2014; Hansson et al., 2015; Moussiliou et al., 2015) involving African child malaria patients showed that *EPCR* gene GG variants are not associated with severe malaria or increased mortality among children with CM. In this study, we examined whether or not the EPCR rs867186-GG genotype is associated with protection against cerebral malaria in Indian malaria patients.

## Methods

Samples used in this study were collected and stored at −80°C as part of an NIH-funded research project (R21TW006804-01) from 2004 to 2007 at the NSCB Medical College Hospital and at the Jabalpur and Civil Hospital Maihar, District Satna, both in the state of Madhya Pradesh, Central India. This study was approved by the ethics research committee of the National Institute of Malaria Research New Delhi, Regional Medical Research Center for Tribals, Jabalpur, Center for Disease Control and Prevention, CDC, Atlanta, GA, and Morehouse School of Medicine, Atlanta, GA, USA.

### Subjects and Enrollment Criteria

Adults and children (< = 14 years) with only *P. falciparum* positive asexual stage parasitemia on blood smear were enrolled after obtaining written informed consent from their parents or close relatives. Consent was also obtained for long-term storage and later use of samples for assessment of biomarkers of disease severity. Patients satisfying the enrollment criteria were enrolled as healthy control (HC), mild malaria (MM), and cerebral malaria (CM) following the definition given below. Malaria associated complications were defined using WHO criteria (Seydel et al., 2015; Sowunmi et al., 2016).

### Healthy Control

Healthy controls (HC) included members of the community who did not have malaria or other febrile illness 60 days before enrollment, and with no history of mental/metabolic illness, tuberculosis, meningitis, or accidental head injury. Thirty-four healthy control samples were left for this current project since the samples used in this study were collected for another project in India in 2007.

### Mild Malaria

Mild malaria group included patients who had a fever with *P. falciparum* parasitemia of <25,000 parasites/μl of blood (detected microscopically from blood smears) and no evidence of severe malaria and no history of mental/metabolic illness, tuberculosis, meningitis, or accidental head injury.

### Cerebral Malaria

Cerebral malaria group included patients who have a Glasgow coma score of ≤10 (Seydel et al., 2015), have a *P. falciparum* parasitemia, and have no other clinically evident cause of impaired consciousness, no history of mental/metabolic illness, tuberculosis, meningitis, or accidental head injury.

### DNA Extraction and rs867186 Genotyping

Genomic DNA was isolated from the peripheral blood mononuclear cells of healthy individuals and malaria patients using the DNeasy Blood and Tissue kit (Qiagen, Valencia, CA).

Primer sequences were designed to contain rs867186 of the *PROCR* gene (NM 006404.3) which were described previously (Moussiliou et al., 2015). The following primers were used to amplify a 660 bp product, forward: CACACGCAGCTTCAGTCAGT; and reverse: TCCCATCCCAAGTCTGACAC (Moussiliou et al., 2015). Genotyping of rs867186 was done by initially amplifying the region of interest using HotStart Taq plus a master mix from Phusion, Cat. no. M0531L (New England Biolabs, Ipswich, MA). Polymerase chain reaction (PCR) conditions are as follows using PCR thermal cycler CFX96 (Bio-Rad, Hercules, CA): 95°C for 15 mins, and then 35 cycles of 95°C for 30 s, 60°C for 40 s, and 72°C for 1 min. PCR products were purified by the kit Cat. no. 28104 (Qiagen, Germantown, MD), and the DNA sequence was determined by Sanger Sequencing (www.Genewiz.com. Genewiz, NJ).

### Estimation of Power for Comparing Two Binominal Proportions

We used two-sided Fisher's exact test to estimate power for comparing two binominal proportions. The significance level of the test is 0.05. Group sample sizes of 23 in the group of mild malaria (MM) and 24 in the group of cerebral malaria (CM) achieved 69.174% power to detect a difference between the group proportions of 0.3600. The proportion in the group of MM is assumed to be 0.0900 under the null hypothesis and 0.4500 under the alternative hypothesis. The proportion in group of CM is 0.0900.

### Statistical Analysis

Chi-Square test or Fisher's exact test were used to compare the rs867189 genotypes in three groups as well as pairwise comparisons. If the expected value of any cell count was less than 5, Fisher's exact test was used. P < 0.05 was considered statistically significant. SAS 9.4 software was used for all the stiatistical test.

## Results

### Clinical Characteristics of Study Subjects

A total of 81 subjects (47 malaria patients and 34 healthy individuals) was included in this study, and the details of patients' clinical characteristics and clinical parameters are shown in **Table 1**. There were no significant differences between patients' age and gender (P > 0.05). Parasitemia is significantly higher in the CM survivor (CMS) group than in the MM group (P = 0.0007). Parasitemia correlated with malaria severity prediction among the MM, CMS, and CM non-survivor (CMNS) groups whereas age and sex did not. The study population comprised of both adults and children, wherein the Glasgow coma score for CMNS (CM mortality rate was 6%) was not significantly different from CMS. Since there are only three cases of cerebral malaria non-survivors (CMNS), we did not analyze them separately. Instead, we analyzed CM patients as the combination of CMS and CMNS. CM patients have significantly higher levels of bilirubin (P = 0.0174) compared to MM patients, which indicated that more severe hemolysis occurred. CM patients presented renal failure more often than MM patients (P = 0.0226), which is evidenced by significantly increased serum urea (P = 0.0039). CM patients suffered from respiratory failure (P = 0.0171) and seizure (P = 0.0040) more frequently than MM patients. Anemia was present in all study groups as compared to HC (Hb = 10.45 ± 2.91). Hemoglobin levels were not significantly different between MM and CMS groups. Thirty-four healthy controls are the community members who were not inflicted with any diseases, no clinical characteristics are available for healthy individuals.

**Table 1:** Clinical characteristics in different malaria types.

		CM	MM	p-value
Gender				0.4745
	Female	8 (33.33)	10 (43.48)	
	Male	16 (66.67)	13 (56.52)	
Seizure				
	No	16 (66.67)	20 (100)	0.0049
	Yes	8 (33.33)	0	
Respiratory Failure				
	No	13 (72.22)	20 (100)	0.0171
	Yes	5 (27.78)	0	
Renal Failure				
	No	16 (66.67)	22 (96.65)	0.0226
	Yes	8 (33.33)	1 (4.35)	
Hypoglycemia				
	No	22 (91.67)	19 (95)	1.0000
	Yes	2 (8.33)	1 (5)	
Hemolysis				0.2389
	No	21 (87.5)	20 (100)	
	Yes	3 (12.5)	0	
DIC				1.0000
		20 (90.91)	18 (90)	
		2 (9.09)	2 (10)	
Age		24.38 (18.95)	24.48 (11.96)	0.9822
Hb		8.00 (2.71)	8.53 (3.06)	0.6124
WBC		7,671 (5,725.8)	4,835.7 (3,461.3)	0.1446
Bilirubin		8.31 (7.54)	0.06 (0.27)	0.0174
Creatinine		3.14 (1.01)	2.11 (2.33)	0.4498
Blood Parasites/µl		15,781.5 (27,232.8)	143.5 (290.9)	0.0007
Urea		92.26 (106.3)	13.35 (40.68)	0.0039

CM, cerebral malaria; MM, mild malaria; DIC, disseminated intravascular coagulopathy; Hb, hemoglobin.

### rs867186 Versus Disease Severity

**Figure 1:**
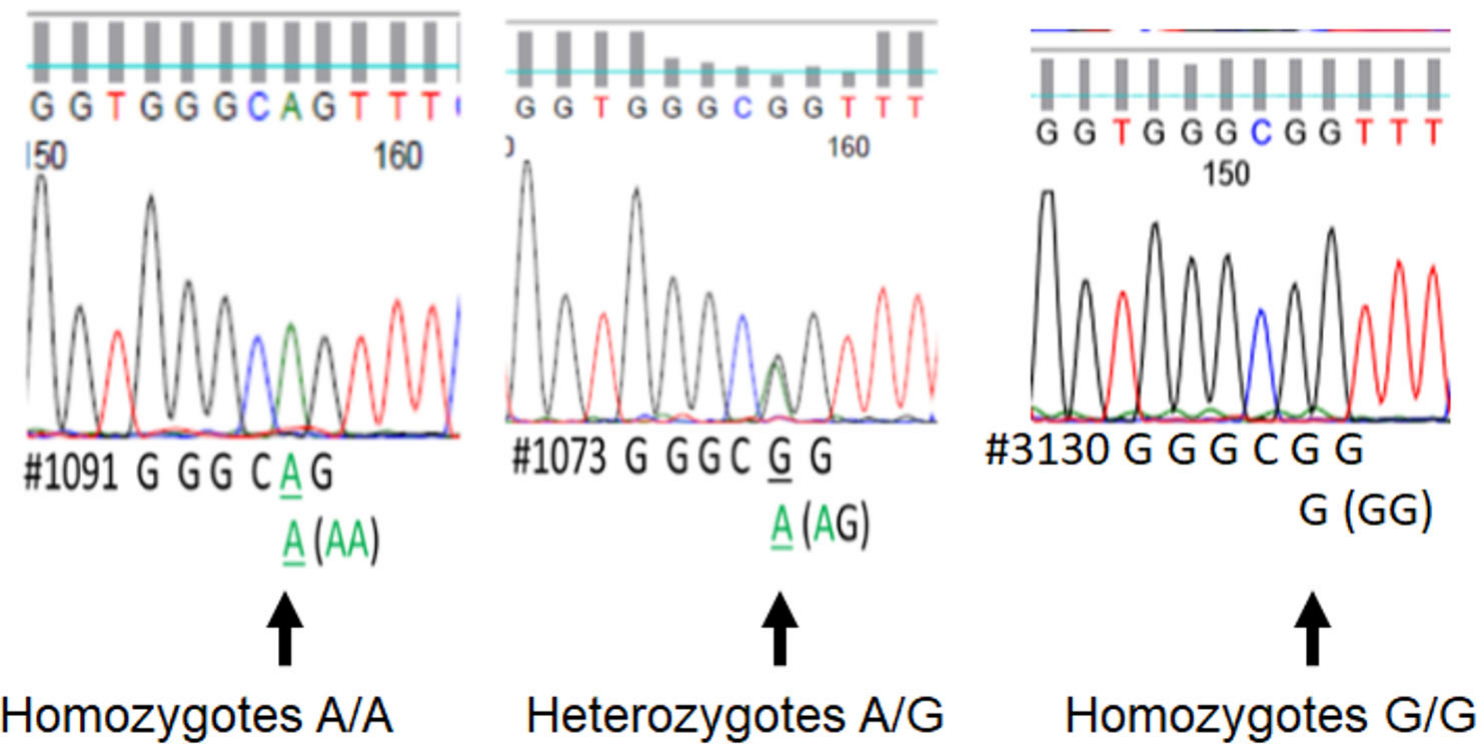
The printout of Sanger sequencing of rs867186 allele from an automated sequencer. The sequence is represented by a series of peaks, one for each nucleotide, a green peak is an “A,” blue is “C,” black is “G,” and red is “T.” It shows the representative image of homozygotes with two copies A (full peak, left), heterozygotes with one copy G/one copy A (half peak, middle), and two copies G (full peak, right), respectively.

We assessed DNA rs867186 polymorphism among 81 subjects. **Figure 1** shows the representative images of homozygotes with two copies A, heterozygotes with one copy G/one copy A, and two copies G respectively. **Table 2** shows the clinical characteristics of different genotypes. Since only two subjects have GG phenotypes among 81 subjects (one is healthy control, and the other is MM patients), we did not compare GG genotypes separately. Instead, we combined AG and GG (AG+GG, G-variant) and compared them to AA genotypes (AG+GG vs. AA). The patients with G-variant (AG+GG) showed higher levels of WBC (P = 0.0042) and hemolysis (P = 0.0263) than patients with AA genotype.

**Table 2:** Clinical characteristics in different genotypes of rs867186 of the *PROCR* gene (EPCR).

		AA	AG+GG	p-value
Gender				0.4053
	Female	14 (38.89)	4 (26.64)	
	Male	22 (61.11)	11 (73.33)	
Seizure				
	No	29 (87.88)	11 (73.33)	0.2358
	Yes	4 (12.12)	4 (26.67)	
Respiratory Failure				
	No	27 (93.10)	10 (76.92)	0.1624
	Yes	2 (6.90)	3 (23.08)	
Renal Failure				
	No	30 (83.33)	12 (80.00)	1.0000
	Yes	6 (16.67)	3 (20.00)	
Hypoglycemia				
	No	30 (90.91)	15 (100)	0.5421
	Yes	3 (9.09)	0	
Hemolysis				0.0263
	No	33 (100)	12 (80.00)	
	Yes	0	3 (20.00)	
DIC				1.0000
		28 (90.32)	14 (93.33)	
		3 (9.68)	1 (6.67)	
Age		24.14 (13.52)	26.67 (20.11)	0.6025
Hb		8.76 (2.85)	7.95 (3.08)	0.4513
WBC		4,714 (3,365.8)	10,896 (5,750)	0.0042
Bilirubin		0.98 (2.37)	0.06 (0.27)	0.1951
Creatinine		2.60 (1.76)	2.23 (1.70)	0.6525
Parasites/µl		5,965.7 (19,470.9)	2,929.7 (6,117.9)	0.5128
Urea		37.80 (81.33)	76.52 (96.95)	0.1781

DIC, disseminated intravascular coagulopathy; Hb, hemoglobin; WBC, white blood cells.

Next we compared the ratios of the rs867186-G variant over -AA genotype in malaria patient groups and healthy controls by 2 ×3 contingency table (**Table 3**). The P-value (P = 0.0172) is highly significant, indicating that some association between the variables is present, therefore, we concluded that the CM patients have higher G-variant (AG+GG) than MM patients is not due to random variation. We then made a pairwise comparison as a *post hoc* analysis for each of the two groups. There are significantly higher rates of rs867186-AG+GG in CM patients compared to mild malaria patients (**Table 4**, P = 0.0044). While the ratio of rs 867186 G-variant over AA genotype did reach significant difference (**Table 5**, P = 0.0221) between MM and HCs, but did not between CM and HCs (**Table 6**).

**Table 3:** The ratio of rs867186 genotype-AA versus -AG and –GG in HC, MM, and CM.

rs867189	HC (%)	MM (%)	CM (%)	Total	
AA	22 (64.7)	21 (91.3)	13 (54.17)	56	
AG+GG	12 (35.3)	2 (8.7)	11 (45.83)	25	
Total	34	23	24	81	P = 0.0172

HC, healthy control; MM, mild malaria; CM, cerebral malaria.

Fisher's Exact Test, Table Probability = 0.0172.

**Table 4:** The ratio of rs867186 genotype-AA versus -AG and -GG in MM and CM.

rs867189	MM (%)	CM (%)	Total	
AA	21 (91.3)	13 (54.17)	34	
AG+GG	2 (8.7)	11 (45.83)	13	
Total	23	24	47	P = 0.0044

MM, mild malaria; CM, cerebral malaria.

**Table 5:** The ratio of rs867186 genotype-AA versus -AG and -GG in HC and MM.

rs867189	HC (%)	MM (%)	Total	
AA	22 (64.7)	21 (91.30)	43	
AG+GG	12 (35.3)	2 (8.70)	14	
Total	34	23	57	P = 0.0221

HC, healthy control; CM, cerebral malaria.

**Table 6:** The ratio of rs867186 genotype-AA versus -AG and -GG in HC and CM.

rs867189	HC (%)	CM (%)	Total	
AA	22 (64.7)	13 (54.17)	35	
AG+GG	12 (35.3)	11 (45.83)	23	
Total	34	24	58	P > 0.05

HC, healthy control; CM, cerebral malaria.

The polymorphism of rs867186 was not affected by age, gender, and parasitemia in this study (P >0.05). Our results indicate that rs867186-G variant (rs867186-GG or rs867186-AG genotypes) are not associated with protection against HCM. The difference in genotype AA and G-variant (AG+GG) of rs867186 is likely due to pressure exerted by parasites of the genus *Plasmodium* that causes malaria. Thus, it may be inappropriate to compare healthy controls that lack of these pressure with any malaria status. This is a possible reason that these genotypes distinguish between mild and severe malaria but not healthy controls and malaria patients.

## Discussion

The surface protein of EPCR was originally cloned in 1994. Multiple ligands of EPCR have been found, including protein C (PC)/activated protein C (APC), factor VIIa, T-cell receptor, and PfEMP1. Under physiological conditions, Protein C is activated by the thrombin-thrombomodulin complex, the activated protein C (APC) then cleaves the protease-activated receptor (PAR1) at Arg46, which triggers anti-apoptotic, anti-inflammatory reaction and inhibits thrombin production and stabilization of endothelial barrier function (Deane et al., 2009; Mohan Rao et al., 2014; Bernabeu and Smith, 2017). In SM, PfEMP1 competitively binds to EPCR with APC and compromises the negative feedback induced by APC, leading to apoptosis, inflammation, loss of endothelial barrier function, localized vascular leakage, and coagulation. Consequently, the microvascular congestion and obstruction exacerbate malaria pathogenesis (Lennartz et al., 2017; Mkumbaye et al., 2017; Tuikue Ndam et al., 2017). Each *P. falciparum* genome holds about 60 PfEMP1-encoding var genes. PfEMP1 containing DC13-variants are capable of binding to both the EPCR (Cham et al., 2009; Lavstsen et al., 2012; Turner et al., 2015; Bernabeu et al., 2016; Jespersen et al., 2016) and ICAM-1 (Howell et al., 2008; Joergensen et al., 2010; Bengtsson et al., 2013; Lennartz et al., 2017), where both are implicated in cerebral malaria. For instance, ICAM-1-binding and rosetting *via* PfEMP1 are the virulence factors for severe malaria (Rowe et al., 2009), while EPCR-binding PfEMP1 dominates host infections with limited malaria immunity (Cham et al., 2009; Lavstsen et al., 2012; Turner et al., 2015; Bernabeu et al., 2016; Jespersen et al., 2016).

The rs867186-GG genotype of EPCR was analyzed in patients with mild malaria and SM by some groups; they found that the rs867186-GG genotype is significantly more frequent in patients with mild malaria than in those with SM (Naka et al., 2014; Shabani et al., 2016). By contrast, our results showed a significantly higher rate of rs867186-G variant (rs867186-GG or-AG) in CM patients compared to mild malaria patients and did not show protection with the increased presence of the G variant. The discrepancy may be due to the racial difference since the subjects in the current study are all Indians; while the studies that showed EPCR rs867186-GG genotypes to be associated with protection against CM had African or Thai subjects. Two studies (Naka et al., 2014; Shabani et al., 2017) that revealed EPCR rs867186-GG genotype could mediate protection against severe malaria only involved adult malaria patients, while the other three studies showed GPCR gene variants were not associated with severe malaria (Schuldt et al., 2014; Hansson et al., 2015) or increased mortality with cerebral malaria involved child malaria patients (Moussiliou et al., 2015). Although they utilized similar criteria for severe malaria, adult and child malaria may have different pathophysiology. In the future, the EPCR polymorphism study should be conducted in adult and child malaria separately.

A soluble EPCR (sEPCR) circulates in plasma and is released from the surface of the endothelium by metalloprotease (Turner et al., 2013); it is a prognostic biomarker for various diseases. In venous thrombosis, Mdina et al. examined the relationship between EPCR polymorphisms and plasma sEPCR levels in patients with thrombosis (Medina et al., 2004). Their data revealed the correlation of the EPCR genotypes 4600AG and 4678CC with high sEPCR and APC levels, as well as a lower risk of venous thromboembolism (Medina et al., 2004). In malaria, loss of endothelial protein C receptor links coagulation and inflammation that are induced by parasite sequestration in cerebral malaria (Moxon et al., 2013; Shabani et al., 2016), and rs867186-GG is associated with increased sEPCR levels and could mediate protection against severe malaria. For instance, a number of research groups found that the rs867186-GG genotype has significantly higher sEPCR levels than those with the AG or AA genotype in SM and healthy individuals, and that the rs867186-AG genotype has significantly higher sEPCR levels than those with AA genotype in uncomplicated malaria. These results suggest that the rs867186 GG genotype is associated with elevated sEPCR levels in SM, and *vice versa*, namely, where sEPCR levels were higher with the increased presence of the G variant. Unfortunately, in the current study, we were unable to examine sEPCR since the matched serum plasma were all used by the primary research project. sEPCR is shed from memberanous EPCR on the surface of the endothelial cells, where it can bind to PfEMP1-DC13 and DC8 variants and free membranous EPCR, (Turner et al., 2013), therefore prevents cerebral malaria. However, the risk of venous thrombosis (Turner et al., 2013; Angchaisuksiri, 2014) would increase due to the increased levels of EPCR. In other words, EPCR protects against SM at the increased risk of thrombotic disease (Turner et al., 2013; Angchaisuksiri, 2014). Therefore, in the context of EPCR polymorphism's protection against SM, balancing the increased risk of thrombotic disease is also worthy of exploration.

In the present study, we analyzed the samples that were collected in 2007 for another project. Although the sample size available for investigations is fixed, an estimate of statistical power with the anticipated available sample size is low but acceptable (see *Methods* section).

## Data Availability Statement

The raw data supporting the conclusions of this article will be made available by the authors, without undue reservation, to any qualified researcher.

## Ethics Statement

Written informed consents were obtained from all participating subjects. This study was approved by the ethics research committee of the National Institute of Malaria Research New Delhi; Regional Medical Research Center for Tribals, Jabalpur; Center for Disease Control and Prevention, CDC, Atlanta, GA, and Morehouse School of Medicine, Atlanta, GA, USA.

## Author Contributions

FY and ML performed statistical analyses. ML and JS conceptualized and wrote the manuscript. JC performed experiments including PCR amplication and PCR product purication for direct sequencing with the help of ML. SK and PB collected blood samples and extracted DNA from human blood samples. ML, JH, and JS participated in the design and coordination of the study. All the authors read and approved the final manuscript.

## Funding

This work was supported by NIH/MIMHD MD007589 and NIH/NINDS R01 NS091616.

## Conflict of Interest

The authors declare that the research was conducted in the absence of any commercial or financial relationships that could be construed as a potential conflict of interest.
